# Socio-demographic and clinical profile of opoid users

**DOI:** 10.1192/j.eurpsy.2024.850

**Published:** 2024-08-27

**Authors:** Z. Bencharfa, R. Zahir, I. El Hamoumi, F. El Omari

**Affiliations:** Ar-razi psychiatric hospital, Salé, Morocco

## Abstract

**Introduction:**

Opioid use disorder is a pattern of problematic opioid use, leading to impaired functioning or clinically significant suffering. Morocco, a pioneer in the Arab world in the field of opiate substitution, is no exception to this rule, and has found itself confronted with a situation where opiate use is much more widespread in the north of the country. Morocco’s geographical proximity to Europe and the multiple interactions fostered by migratory population flows undoubtedly contribute not only to the spread of hard drug use, particularly heroin, but also to the diversification of consumption methods (injection drugs)

**Objectives:**

The main objective of our work was to study socio-demographic and clinical profile of opoid users in morocco, but also their quality of life after treatment in Morocco, before concluding with recommendations for improving the overall management of the patient.

**Methods:**

We conducted a cross-sectional, analytical study in the Addictology Department at Ar-razi Hospital in Salé, which provides oral methadone substitution therapy for around 80 patients.

**Results:**

The total number of patients responding to the questionnaire was 60 participants.

The population of methadone-treated patients in our study was 83.33% (n = 50) male and 16.67% female (n = 10).

The most common age group in our study was between 31 and 45 (71.67%). 36.67% were married (n=22), 80% (n=48) lived with their family, 83.34% (n=50) had a secondary school education or higher, while the vast majority 63.33% (n=38) had no fixed occupation.

96.7% (n=58) of participants were of Moroccan nationality, against only 3.3% who were foreigners (n=2).

The main indication for methadone withdrawal in our patients was heroin use (66.67%), followed by Codeine, then Tramadol. The daily doses of methadone delivered ranged from 04 to 200 mg/patient, with an average of 75 mg.

The main adverse effects reported by our patients were libido disturbance, constipation, fatigue and sleep disturbance.

63.33% (n=38) of patients continued to use other psychoactive substances on a regular basis, mainly tobacco, followed by cannabis.

13.33% (n=8) reported persistent craving, and the vast majority claimed to be supported by a family member (70%, n=40).

**Conclusions:**

For several years, quality of life has been a major preoccupation of healthcare professionals in a bio-psycho-social approach. In this vision of care, quality of life should now be part of the clinical criteria for monitoring patients on methadone.

**Disclosure of Interest:**

None Declared

